# Association between maternal human immunodeficiency virus infection and preterm birth: A matched case-control study from a pregnancy outcome registry

**DOI:** 10.1097/MD.0000000000022670

**Published:** 2021-01-29

**Authors:** Narcisse Elenga, F.é.lix Djossou, Mathieu Nacher

**Affiliations:** a Pediatric Medicine and Surgery; b Tropical and Infectious Disease Unit; c Centre d’Investigation Clinique Antilles-Guyane, Inserm 1424, Andrée Rosemon Regional Hospital, Rue des flamboyants, Cayenne Cedex, French Guiana.

**Keywords:** French Guiana, human immunodeficiency virus-positive women, preterm birth, protease inhibitor-based antiretroviral therapy

## Abstract

This study aimed to evaluate the relationships between different types of antiretroviral therapy (ART) and preterm birth.

Preterm birth was studied among all singleton pregnancies and compared between human immunodeficiency virus (HIV)-infected and uninfected women.

We performed a matched case-control study from the pregnancy outcome registry of Cayenne Hospital. HIV-infected and uninfected women who delivered in the maternity ward of Cayenne Hospital from January 1, 2013 to December 31, 2015 were studied. We conducted an initial analysis to determine the risk factors for preterm birth among HIV-infected pregnant women. We also evaluated associations between exposure to antiretroviral therapy (ART) and preterm birth.

There were 8682 deliveries; of these, 117 involved HIV-infected women, representing a prevalence of 1.34%. There were 470 controls. The sociodemographic characteristics were comparable. HIV-infected women were more likely to experience preterm birth (adjusted odds ratio [AOR] = 3.9, 95% confidence interval [CI] 1.5–9.9). Overall, 95.73% of the women received antiretroviral therapy before becoming pregnant, and they were in good clinical condition. The median CD4 count at the beginning of pregnancy was 500 cells/mm^3^ (357–722). Additionally, 53% of HIV-infected women had an undetectable viral load count (<20 copies/mL). Their median haemoglobin level was 120 g/L (100–120). There were 2 human immunodeficiency virus-infected babies. A higher rate of preterm birth was associated with protease inhibitor-based ART than a reverse transcriptase inhibitor-based ART regimen. The sample size being small this result would be considered with caution.

The preterm birth rate among HIV-infected pregnant women was twice that of the general population; this trend was not explained by sociodemographic characteristics. Preterm birth was independently associated with combination ART, especially with ritonavir-boosted protease inhibitor therapy during pregnancy.

## 1 Introduction

There are 36.7 million people worldwide living with the human immunodeficiency virus (HIV) (34 million–39.8 million), and the annual incidence of HIV is 2.1 million.^[1]^ Recent advances in antiretroviral therapies (ARTs) have improved survival and quality of life for HIV-infected individuals. This prolonged survival will contribute to an increase in pregnancies among HIV-infected women. At the end of 2015, approximately 77% (69%–86%) of pregnant HIV-infected women had access to ARTs to prevent HIV transmission to their babies. The antiretroviral regimens used to prevent mother-to-child transmission of HIV have evolved from the first successful trial that used Zidovudine single-drug prophylaxis in 1994 to the current triple-drug regimens.^[2]^ Although there are clear benefits of ART combinations for the mother and infant, these treatments do have risks, and some studies have shown higher rates of adverse pregnancy outcomes with maternal ART combinations than with regimens containing fewer antiretroviral agents.^[3,4]^

French Guiana is a French territory in South America with an HIV prevalence exceeding 1% among pregnant women.^[5]^ Among the HIV-infected people who were followed in Cayenne Hospital in 2011, 76.6% were foreigners.^[6]^ The standard of care for HIV-infected people is the same as for any other French territory (medical follow-up with specialized infectious disease physicians and routine viral load and CD4 monitoring every 3 to 6 months). Since September 2013, anti-retroviral drugs have been offered to any HIV-infected people, according to French guidelines.^[7]^ From 2013 to 2015, all HIV-infected pregnant women were treated according to the national protocol, which, regardless of the CD4 count or the viral load, recommends highly active antiretroviral therapy (HAART) using 2 nucleoside-reverse-transcriptase inhibitors (NRTIs) and a boosted protease inhibitor (PI) from the beginning of pregnancy until delivery.^[7]^ All children were followed for 24 months to determine their HIV status and to detect any developmental abnormalities. Breastfeeding was contraindicated for all HIV-infected mothers. In French Guiana, since 2 decades, the proportion of premature birth is around 13.5%, which is nearly the double of that reported in mainland France. Given that persistent high rate of prematurity, additional studies are needed to evaluate possible risk factors for preterm birth, including ARTs.

Although studies have documented associations between maternal HIV infection and adverse pregnancy outcomes, conflicting results have been found for the association between preterm birth and ART.^[8–10]^ Previous studies often had limited power, lacked comparison groups and involved populations from geographically disparate areas with varying degrees of access to treatment and different drug treatment regimens. Thus, our study aimed to examine in a French region with a high preterm birth rate, the effects of maternal HIV status and maternal ART on preterm delivery.

Our primary outcome was the effect of HIV status on preterm birth (birth before 37 weeks of pregnancy). Our secondary outcomes were the effects of HIV status on other maternal and foetal outcomes such as intrauterine growth restriction (IUGR) (neonates born with clinical features of malnutrition and in-utero growth retardation, irrespective of their birth weight percentile) and the relationships between ART type and preterm birth.

## 2 Materials and methods

### 2.1 Study site

The study was conducted in Cayenne General Hospital, the only type 3 maternity in French Guiana.

### 2.2 Data collection

The exhaustive data from the delivery registry RIGI (Registre Issue des Grossesses Informatisé), managed by the réseau perinat (a network of health professionals in perinatality in French Guiana) was studied for a period of 3 years, from January 1, 2013 to December 31, 2015. The ultrasound (US) performed within 10 to 13 weeks and 6 days of pregnancy was considered the method used to estimate the gestational age, when women entered the registry.

### 2.3 Sample size calculation

Considering that the prevalence of prematurity is 13.5% in French Guiana. The sample size was calculated with the hypothesis that the rate of preterm birth is twice more in HIV infected women. Sample size was calculated based on power of 0.80 (Power = 1–*β*) while probability of type I error (*α*) was set at 0.05; with a one-tailed test. The minimum sample size required based on this hypothesis was 114 for the cases and 570 for controls.

### 2.4 Type of study

The study was a matched case-control study based on the pregnancy outcome registry of Cayenne Hospital. We have included in our study only single deliveries, of living children. The exhaustive data from this registry are routinely entered at the time of delivery according to the patient's medical and obstetrical history (medical records and interviews). Multiple variables are recorded in the registry: sociodemographic, all pathologies associated with pregnancy, the term, place of birth, the mode of labor initiation, the sex, weight, height, cranial perimeter, Apgar, malformations, child outcomes… All viable births occurring after 22 weeks of amenorrhea were included. HIV-infected and uninfected women who delivered in the maternity ward of Cayenne General Hospital from January 1, 2013 to December 31, 2015 were included in the study.

The cases were HIV-infected mothers who delivered at Cayenne Hospital between January 1, 2013 and December 31, 2015. All these patients were also included in the CO1-EPF, which is a French prospective observational cohort study of mother-child pairs and infected and uninfected children who have been enrolled since 1986.^[11]^

The controls were HIV-uninfected mothers who delivered at Cayenne Hospital between January, 1, 2013 and December 31, 2015. All women having agreed to participate in the study were included. Women who delivered at home in Cayenne or during transport were also included if they fit the inclusion criteria. DNA PCR or RNA PCR were used to diagnose HIV infection in neonates and infants under 18 months of age who are born of HIV positive mothers. In addition, HIV serology was performed after 18 months to allow the mandatory reporting of HIV infection to French Institute for Public Health Surveillance, as recommended in France.

### 2.5 Identification of cases

From the French Guianese pregnancy registry and the computerized HIV patient file eNADIS,^[12]^ we identified cases according to the above inclusion criteria. All HIV-infected women who gave birth during the study period were included (cases).

### 2.6 Identification of controls

From the registry, women were randomly sampled in order to obtain 4 controls per case, matched by year of delivery.

Exclusion criteria were as follows: age under 18, refusal to participate in the study. We also excluded from the study stillbirth, abortion, and multifetal gestations.

### 2.7 Data collection

The following data were collected from several sources (i.e., pregnancy registry, eNADIS, patient medical records, and the medical information system): sociodemographic data (i.e., residence, age, birthplace, education level, possession of health insurance, marital status, and profession) and obstetrical factors (i.e., parity, gravidity, pathologies during pregnancy, mode of delivery, birth term, condition of the child and mother at birth, and child HIV serology after 18 months of age).

### 2.8 Data analysis

The data were entered into Microsoft Excel 2007 and analyzed using Stata 13 (Stata Statistical Software: Release 13. College Station, TX: Stata Corp LP). Quantitative variables were categorized according to statistical criteria using the first and third quartiles and the median. Single comparisons were performed using Student's *t* test for quantitative variables and the CHI2 or Fisher exact test for qualitative variables. Bivariate analysis was used to study covariates and their relationships with the outcome measures based on the crude odds ratio and its confidence interval. The covariates that were associated with the outcomes were then included in an unconditional multivariate logistic regression model. We expressed the results as the means, odds ratios, and confidence intervals. *P*-values <.05 were considered to be statistically significant. A priori, the following variables were considered to be potential confounders, and all models were adjusted for maternal age, birthplace, occupation, parity, and immunovirological status. We conducted an initial analysis to determine the risk factors for preterm birth among HIV-infected pregnant women. Second, we conducted a separate analysis to evaluate the potential association between ART exposure and preterm birth. Pearson goodness of fit test was used.

### 2.9 Ethical and regulatory aspects

The Commission Nationale Informatique et Libertés (CNIL), which is a national committee that oversees research data, approved the collection of this anonymized data from the French Guianese pregnancy registry.

### 2.10 Follow-up and ART strategies

In French Guiana, our recommendation was to offer to all women living with HIV-1 a long course treatment, started as early as possible and continued after delivery. During the study period, the first choice was a triple therapy comprising 2 NRTIs and a PI associated with ritonavir (PI/r). The combination of zidovudine + lamivudine had the greatest favorable clinical experience and remained a first-line option despite its known toxicities. Another first-line option, which was as widely prescribed as tenofovir, usually in combination with emtricitabine. Regarding PI/r, we favored those for which there was clinical data: lopinavir/r and atazanavir/r which were the most studied. Although pregnancy would result in a significant decrease in plasma concentrations of PI, in the 3rd quarter; we preferred to recommend a standard dose of IP/r, as well as the INRT.

Obviously, when the treatment was ineffective, it was recommended to adapt to the genotype of resistance, if necessary using little known molecules during pregnancy. Monitoring a pregnant woman on antiretroviral therapy involved measuring plasma HIV RNA every month to assess compliance and treatment, the assay of CD4 lymphocytes once per quarter, and assessment of biological tolerance to ARVs every 2 months. The viral load at 34 to 36 weeks of gestation makes it possible to decide on the mode childbirth.

Despite free testing and treatment, HIV pregnant women in French Guiana, often have delayed or insufficient access to care, especially for foreign women in an irregular situation. These women can sometimes represent a high proportion.^[13]^ This is why our strategy recommends vaginal delivery and post-natal treatment with nevirapine or zidovudine if the maternal treatment before delivery is >8 weeks and the viral load at delivery is undetectable, and elective caesarean section with zidovudine maternal infusion, and intensified postnatal neonatal treatment with nevirapine, zidovudine, and lamivudine if the viral load at delivery is >400 copies/mL. Other medications such as a daily combined tablet containing iron and folic acid have been prescribed for HIV +pregnant women, but also for controls.

## 3 Results

From 2013 to 2015, there were 8682 deliveries, and of these deliveries, 117 were HIV-positive women, which resulted in a prevalence of 1.34% of deliveries. There were 470 controls. The pregnancy registry showed that 99% of HIV-positive women in the Cayenne area delivered at this hospital. All but 5 of these women had initiated ART before conception or during the first trimester. They all were followed by the specialized infectious disease physicians and addressed very early for antenatal care.

Table 1 shows the baseline characteristics of the women according to their HIV status (missing data were not included in the analyzes). Compared to uninfected women, women infected with HIV tended to be older in age (odds ratio [OR] = 1.6 [1.3–2.1], *P* < .001) and were more likely to be born in Haïti (OR = 1.5 95% CI 1.13–1.9, *P* = .03), to have a high parity (OR = 2.9 95% CI 1.8–4.5, *P* < .001) and to have no occupation (OR = 5.8 95% CI 2.3–14.6, *P* < .001). HIV-positive women also had a comparatively greater frequency of abnormal foetal heart rate (OR = 12 95% CI 3.8–38.5, *P* < .001). There were no differences in the risks for other complications by HIV status. There were no cases of suspected primary hyperaldosteronism (PHA) nor trauma. However crack cocaine use concerned <1% of pregnant women.

**Table 1:** Characteristics according to the patients’ HIV status.

	HIV+	HIV–	
Characteristics	n = 117	n = 470	*P*
Median age (years, IQ)	33 [27–37]	28 [23–34]	**<.001**
Birth place			**.003**
France	23 (20)	204 (44)	
Haiti	49 (44)	111 (24)	
Others	40 (36)	150 (32)	
Status			.4
Living common law	39 (33)	185 (40)	
Single	22 (19)	58 (13)	
Not specified	56 (48)	217 (47)	
Social security			.1
SS or Universal health coverage	33 (43)	247 (64)	
State medical aid	3 (4)	21 (5)	
No SS	10 (13)	33 (9)	
Not specified	31 (40)	85 (22)	
Occupation			**<.001**
Entrepreneur, executive	1 (1)	7 (3)	
Employee, student	3 (4)	81 (29)	
No occupation	68 (95)	189 (68)	
Median parity (IQ)	3 [3–4]	2 [1–3]	**<.001**
Gestational diabetes			.2
Yes	1 (1)	14 (3)	
No	116 (99)	454 (97)	
Arterial hypertension			.8
Yes	1 (1)	5 (1)	
No	116 (99)	463 (99)	
Preeclampsy			.5
Yes	1 (1)	8 (2)	
No	116 (99)	460 (98)	
Preterm premature rupture of the membranes	10 (9.3)	40 (9.8)	.8
Placenta previa	1 (0,9)	3 (0,7)	.6
Abnormal fetal heart rate			**<.001**
Yes	1 1 (9)	4 (1)	
No	106 (91)	464 (99)	
Intrauterine growth retardation			.9
Yes	6 (5)	9 (5)	
No	111 (95)	174 (95)	
Fetal death			.6
Yes	2 (2)	5 (1)	
No	115 (98)	463 (99)	

Missing data were not included in the analyzes. Bold values signify statistically significant.

Table 2 displays the association between HIV status and antepartum complications. HIV-positive women were more likely to experience preterm birth (AOR = 3.1, 95% CI 1.7–5.7). This preterm birth was moderate, with a median gestational age of 34.5 weeks of amenorrhea (WA).^[32–34]^ HIV infection was not associated with a greater risk of IUGR.

**Table 2:** Association between HIV status and antepartum complications.

	HIV+	HIV–		
Characteristics	n (%)	n (%)	AOR	*P* ^∗^
Intrauterine growth retardation			0.9 [0.3–3]	.9
Yes	6 (5)	9 (5)		
No	111 (95)	174 (95)		
Fetal death			0.3 [0.1–1.5]	.1
Yes	2 (2)	5 (1)		
No	115 (98)	463 (99)		
Median parity (IQ)	3 [3–4]	2 [1–3]		
Abnormal fetal heart rate				
Yes	1 1 (9)	4 (1)	3.9 [1.2–13.9]	**.03**
No	106 (91)	464 (99)		
Preterm birth			3.1 [1.7–5.7]	**<.001**
Yes	37 (32)	63 (13)		
No	80 (68)	405 (87)		

AOR = adjusted odds ratio.

∗ *P* value of multivariate analysis.

With respect to the HIV-positive women, 95.73% of them received ART before becoming pregnant, and they were in good clinical condition. No HIV-infected woman, among those in our study, had an opportunistic infection or tuberculosis. In addition, 1% of them were coinfected with hepatitis B. No significant side effects related to antiretroviral drugs have been noticed. The median CD4 count of the HIV-positive women at the time of delivery was 500 cells/mm^3^ (357–722). Overall, 53% of the HIV-positive women had an undetectable viral load count (<20 copies/mL) at the time of delivery. Among the women with detectable viral loads, the median viral load count was 1518 copies/mL (246–3324). The median hemoglobin level was 120 g/L (100–120), and only 10% of the HIV-positive women had a hemoglobin concentration <100 g/L. This rate of anemia was identical in the controls. There were 2 HIV-infected babies, including 1 baby born to a woman who did not receive ART. The second infected baby was born with cerebral toxoplasmosis complicated by microcephaly.

Table 3 shows the association between antiretroviral therapy and preterm birth. This result demonstrated that there was a higher rate of preterm birth associated with PI-based ART than RTI-based ART regimens.

**Table 3:** Association between antiretroviral therapy and preterm birth.

		Term birth	AOR	
Characteristics	Preterm birth	n (%)	n (%)	*P*
Antiretroviral therapy			4.4 [1.2–15.4]	.02
RTI-ART regiment	6 (14.6)	35 (85.4)		
PI-ART regiment	31 (44.9)	38 (55.1)		
Hb level	12 [10.5–13]	12 [10–12]	1.5 [0.22–9.8]	.7
CD4	500 [387.5–746.5]	488.5 [357–722]	0.7 [0.30–1.8]	.5
Viral load (if detectable)	1518 [328–2325]	1516 [163.5–7536]	1.2 [0.42–3.4]	.7

AOR = adjusted odds ratio; PI-ART regimen = protease inhibitor antiretroviral therapy regimen; RTIs-ART regimen = reverse transcriptase inhibitor antiretroviral therapy regimen.

## 4 Discussion

In our study, the majority of HIV-infected pregnant women received ART before pregnancy. These women were in good general condition and had good immune restoration, with few comorbidities and rare cases of anemia. For those who still had a detectable viral load, the viral load was not very high. The overall health of the HIV-infected women appeared to be comparable to uninfected women. Indeed, the primary goal of ART in HIV-infected women before or during pregnancy is to prevent both HIV-associated morbidity and mortality and perinatal transmission of HIV. As shown in other published studies from French Guiana, the majority of HIV-infected pregnant women were foreigners.^[6]^ These results are similar to those reported in the French Perinatal Cohort.^[14]^ In our study, the overall premature birth rate was 13.5% in HIV-uninfected women and 31.6% in HIV-infected women. This rate was markedly higher than the rate reported for the French Guianese general population (13.5%). Similarly, high preterm birth rates among HIV-exposed children have been found in numerous studies.^[4,15–17]^ It has been previously reported that compared with unexposed children, children who encounter intrauterine HIV exposure are at high risk of low birth weight (LBW).^[18]^ In addition, there were no substantial differences in these associations between developing and developed countries. Remarkably, we observed no significant difference in LBW rates between HIV-infected women and uninfected women. Pregnancy affects every system in the body. Changes in hormone levels and immune function can increase the susceptibility of pregnant women to certain infections and serious complications. These fluctuations in hormone levels often affect the urinary tract. As the uterus expands during pregnancy, it puts more pressure on the ureter. Meanwhile, the body increases the production of progesterone, which relaxes the ureter and bladder muscles. As a result, urine may stay in the bladder too long. This change increases the risk of developing a urinary tract infection. Hormonal changes also make pregnant women more susceptible to a type of yeast infection known as candidiasis. Higher levels of estrogen in the reproductive tract predispose women to yeast infections. If accompanied by HIV infection, disease progression can be accelerated. HIV-associated preterm birth might be related to progressive immunodeficiency marked by CD4T lymphocyte (CD4) cell depletion. Previous studies showed that women with CD4 cell counts <350 had a high risk of LBW and preterm birth.^[4,8,9,17]^

Some studies have reported that HIV-1 can replicate in the placenta,^[19]^ and that HIV-1 infection may alter the cytokine profile in the placenta.^[20,21]^ This alteration may affect the function of the placenta during pregnancy and restrict foetal development, which maybe another cause of preterm birth. In addition, the activation of the maternal and/or foetal hypothalamo-pituitary-adrenal (HPA) axis by maternal and/or foetal stress, inflammation (systemic or decidual chorioamniotic), decidual hemorrhage, and pathological distention of the uterus could be other pathogenic pathways for preterm birth.^[22]^ It has also been demonstrated that psychological and/or social stress may be a significant independent risk factor for preterm birth.^[23]^ Under certain circumstances such as HIV infection, the pregnancy itself can become a stressful and difficult life experience. Stress during pregnancy can also affect fetal heart rate, as seen in our results and in previous studies.^[24]^

Our results demonstrated the relationship between PI-based ART use during pregnancy and preterm birth. The relationship between PI use during pregnancy and preterm birth has been of interest for the past 10 years. Initial studies examining this association found an increased risk of preterm birth with the use of combination antiretrovirals (cARV) with PIs in comparison to ARV without PIs.^[14,25–27]^ In contrast, in the United States, apart from 2 studies,^[28,29]^no such relationship was found.^[30–32]^ These conflicting results may be attributable to variability in the number of patients studied, differences between the study populations, and differences in the age distribution, as well as the drug regimens that were used.

The main strength of the current study is that our study focused on a comparable population, with no many sociodemographic differences between the PI group and the non-PI group. The majority of pregnant women began routine standard of care ARV therapy before or during pregnancy. In addition, there was homogeneity in the therapeutic combinations that were used. The following 2 modalities were used: ART with PI and ART without PI. All the PIs that were used were boosted by ritonavir. We adjusted our analysis according to factors that are known to be associated with preterm birth in the general population. We also attempted to remove residual confounding factors in the study population through the addition of hemoglobin levels, CD4+ cell counts, and viral loads to our multivariate models.

Our most important finding was a significant association between preterm birth and ritonavir-boosted PI therapy in comparison to non-PI-based therapy. Our results may be directly comparable to previous analyses that found associations between PI use and preterm birth. Ritonavir alone could not explain this result. Indeed, it has been reported that ritonavir is associated with complex metabolic changes and could interfere with the adrenal systems of both the mother and the foetus through its interactions with CYP3A4.^[33]^ These adrenal systems are involved in the spontaneous onset of labor,^[34]^ and ritonavir-boosted PIs may influence its timing.

Our study has some limitations. Due to the selection criteria, our numbers are smaller than those used in some previous published studies. In addition, the number of women on ART without PI is relatively small, and all the women who received regimens without PI still received highly active regimens. The sample size was too small to suggest generalizable results. We cannot rule out differences caused by the PI, lopinavir or another PI, rather than the ritonavir boost.

## 5 Conclusion

Among HIV-infected pregnant women, the preterm birth rate was twice that in the general population in French Guiana, and this difference was not entirely explained by sociodemographic characteristics. Preterm birth was independently associated with ART, especially with the initiation of ritonavir-boosted PI therapy during pregnancy. Although a causal relationship cannot be confirmed, our findings suggest that there is a plausible explanation for the association between ART and preterm birth that merits further investigation. Boosted PI therapies are the standard of care during pregnancy; therefore, this study may have important clinical implications.

## Acknowledgments

The authors would like to thank Dr Josiane Warszawski from the INSERM U1018 department of Paris-sud 11 University in France for her advice and corrections.

## Author contributions

**Conceptualization:** Narcisse Elenga.

**Data curation:** Narcisse Elenga.

**Formal analysis:** Narcisse Elenga.

**Methodology:** Narcisse Elenga, Mathieu Nacher.

**Supervision:** Narcisse Elenga, Félix Djossou, Mathieu Nacher.

**Validation:** Félix Djossou.

**Writing – original draft:** Narcisse Elenga.

**Writing – review & editing:** Narcisse Elenga, Félix Djossou, Mathieu Nacher.

## Abbreviations

Abbreviations: AOR = adjusted odds ratio, ART = antiretroviral therapy, ARV = antiretroviral, cARV = combination antiretrovirals, CNIL = Commission Nationale Informatique et Libertés, HAART = highly active antiretroviral therapy, HIV = human immunodeficiency virus, HPA = hypothalamo-pituitary-adrenal, IUGR = intrauterine growth restriction, LBW = low birth weight, NRTIs = nucleoside-reverse-transcriptase inhibitors, OR = odds ratio, PHA = primary hyperaldosteronism, PI = protease inhibitor.
